# Temporal Profile and Mechanisms of the Prompt Sympathoexcitation following Coronary Ligation in Wistar Rats

**DOI:** 10.1371/journal.pone.0101886

**Published:** 2014-07-09

**Authors:** Luciana Mesquita Passamani, Ana Paula Abdala, Davi José de Almeida Moraes, Karla Nívea Sampaio, José Geraldo Mill, Julian Francis Richmond Paton

**Affiliations:** 1 School of Physiology & Pharmacology, Bristol Heart Institute, Medical Sciences Building, University of Bristol, Bristol, United Kingdom; 2 Department of Physiological Sciences, Health Sciences Center, Federal University of Espírito Santo, Vitória, Espírito Santo, Brazil; 3 Department of Physiology, School of Medicine of Ribeirão Preto, University of São Paulo, Ribeirão Preto, São Paulo, Brazil; 4 Department of Pharmaceutical Sciences, Health Sciences Center, Federal University of Espírito Santo, Vitória, Espírito Santo, Brazil; University of Central Florida, United States of America

## Abstract

Our aim was to assess the timing and mechanisms of the sympathoexcitation that occurs immediately after coronary ligation. We recorded thoracic sympathetic (tSNA) and phrenic activities, heart rate (HR) and perfusion pressure in Wistar rats subjected to either ligation of the left anterior descending coronary artery (LAD) or Sham operated in the working heart-brainstem preparation. Thirty minutes after LAD ligation, tSNA had increased (basal: 2.5±0.2 µV, 30 min: 3.5±0.3 µV), being even higher at 60 min (5.2±0.5 µV, P<0.01); while no change was observed in Sham animals. HR increased significantly 45 min after LAD (P<0.01). Sixty minutes after LAD ligation, there was: (i) an augmented peripheral chemoreflex – greater sympathoexcitatory response (50, 45 and 27% of increase to 25, 50 and 75 µL injections of NaCN 0.03%, respectively, when compared to Sham, P<0.01); (ii) an elevated pressor response (32±1 versus 23±1 mmHg in Sham, P<0.01) and a reduced baroreflex sympathetic gain (1.3±0.1 versus Sham 2.0±0.1%.mmHg^−1^, P<0.01) to phenylephrine injection; (iii) an elevated cardiac sympathetic tone (ΔHR after atenolol: −108±8 versus −82±7 bpm in Sham, P<0.05). In contrast, no changes were observed in cardiac vagal tone and bradycardic response to both baroreflex and chemoreflex between LAD and Sham groups. The immediate sympathoexcitatory response in LAD rats was dependent on an excitatory spinal sympathetic cardiocardiac reflex, whereas at 3 h an angiotensin II type 1 receptor mechanism was essential since Losartan curbed the response by 34% relative to LAD rats administered saline (P<0.05). A spinal reflex appears key to the immediate sympathoexcitatory response after coronary ligation. Therefore, the sympathoexcitatory response seems to be maintained by an angiotensinergic mechanism and concomitant augmentation of sympathoexcitatory reflexes.

## Introduction

Coronary artery ligation is accepted to mimic myocardial infarction (MI) causing sympathetic nervous system over activity [1], [2]. Although the enhanced sympathetic activity provides initially inotropic support to the heart, maintaining cardiac output and arterial pressure following MI, it is also associated with the pathogenesis of ventricular arrhythmias [3]–[5] and sudden cardiac death [6]. Further, the sustained sympathoexcitation may contribute to the cardiac pathology leading to progressive cardiac remodelling and development of heart failure [7]–[9]. Thus, a thorough understanding of timing of the sympathoactivation and any change in the mechanisms for it may be important for improving future treatment strategies, e.g. the appropriate timing of medication to antagonise sympathetic effects on the vasculature versus the heart may be essential for maintaining arterial pressure but preventing cardiac arrhythmias/sudden cardiac death.

Most of our knowledge on changes in cardiovascular autonomic balance has been obtained days after MI or later in the course of chronic heart failure, using indirect methodological approaches such as measuring plasma catecholamine levels [10], heart rate variability (HRV) and cardiovagal baroreflex sensitivity [11]. Little information about the time course and contributing mechanisms of sympathetic changes immediately following a MI exists (i.e. acute phase). Sequential and direct measurements of sympathetic drive are essential to elucidate these issues. In this regard, a number of chronic studies have shown a multifactorial process for sympathetic modulation after MI, which may include: cardiac vagal and cardiac sympathetic afferents [12], [13], changes in peripheral neural reflexes (peripheral chemoreceptor reflex) [14], sympathetic neural remodelling [15] and activation of brain angiotensinergic mechanisms [16]. However, the importance and sequence of activation of these mechanisms immediately following MI have not been characterized. Our hypothesis was that there would be a sustained and immediate (within 30 min) increase in sympathetic activity and reduction in cardiac vagal tone post coronary ligation and that multiple time-dependent mechanisms underpin the sympathoexcitation. Thus, the purpose of this study was to determine when the autonomic imbalance occurred after ligation of the left anterior descending coronary artery (LAD) to mimic MI, and the underlying mechanisms.

## Methods

### Animals

Experiments were performed on male Wistar rats (60–100 g) using an anaesthetic-free preparation with a preserved functional brainstem – the working heart-brainstem preparation (WHBP) [17], [18]. All surgical and experimental procedures were carried out in compliance with the United Kingdom Animals (Scientific Procedures) Act 1986 and were approved by the University of Bristol Animal Ethics Committee (Permit number: PPL 30/3121).

### WHBP and nerve recordings

Animals were pre-treated with heparin sodium (1000 units, i.p.) and deeply anaesthetized with isoflurane (5%, vaporized in 95% O_2_−5% CO_2_), until loss of the paw withdrawal reflex. Following sub-diaphragmatic transection, rats were exsanguinated and anaesthesia terminated. Preparations were submerged in ice-chilled Ringer solution (composition in mM: 1.25 MgSO_4_.7H_2_O, 1.25 KH_2_PO_4_, 3.0 KCl, 25.0 NaHCO_3_, 125.0 NaCl, 2.5 CaCl_2_.2H_2_O and 10 D-glucose), decerebrated pre-collicularly and a 6.0 suture passed around LAD. The preparation was placed into a recording chamber and a double-lumen catheter inserted into the descending aorta for retrograde perfusion. The second lumen of the catheter was used to monitor perfusion pressure (PP). The perfusate was carbogen gassed (to pH 7.4), warmed {31°C; which was optimal for viability as originally described by Paton [18]} Ringer solution containing Ficoll 70 (1.25%), which was recirculated using a peristaltic roller pump. Neuromuscular blockade was established using vecuronium bromide (2 mg/mL; Organon). Mean PP was >60 mmHg achieved by adjusting flow rate (20–22 mL/min) and addition of arginine vasopressin (20–60 µL of 5 µM; Sigma). Heart rate (HR) was derived from the ECG. The left phrenic and thoracic sympathetic nerves (T8–T10) were recorded simultaneously with bipolar suction electrodes. Minutes after the onset of perfusion an augmenting (i.e. eupnoeic) pattern of phrenic nerve activity was achieved. All signals were amplified, band-pass filtered (0.5–5 kHz) and acquired using a CED 1401 A/D Converter and analyzed using Spike2 software (Cambridge Electronic Design).

### Coronary ligation in situ and measurement of area at risk

After baseline recordings of phrenic (PNA) and thoracic sympathetic nerve activities (tSNA), HR, ECG and PP, either LAD ligation was carefully performed without affecting nerve recordings; or the suture remained untied in Sham operated preparations. At the end of each experiment, the heart was rapidly excised and via the ascending aorta perfused with 1% Evans blue dye (Sigma) to indicate the myocardial area at risk as reported previously [19], [20]. Once perfusion was completed, the atria were removed. After overnight fixation in 10% formalin, the ventricles were cut in five transverse slices and digitally photographed and analyzed using ImageJ software. The area at risk was determined as the percentage of Evans blue non-stained area relative to the entire left ventricle area. In all Sham operated rats, no cardiac area at risk was observed.

### Experimental protocols

**Figure 1:**
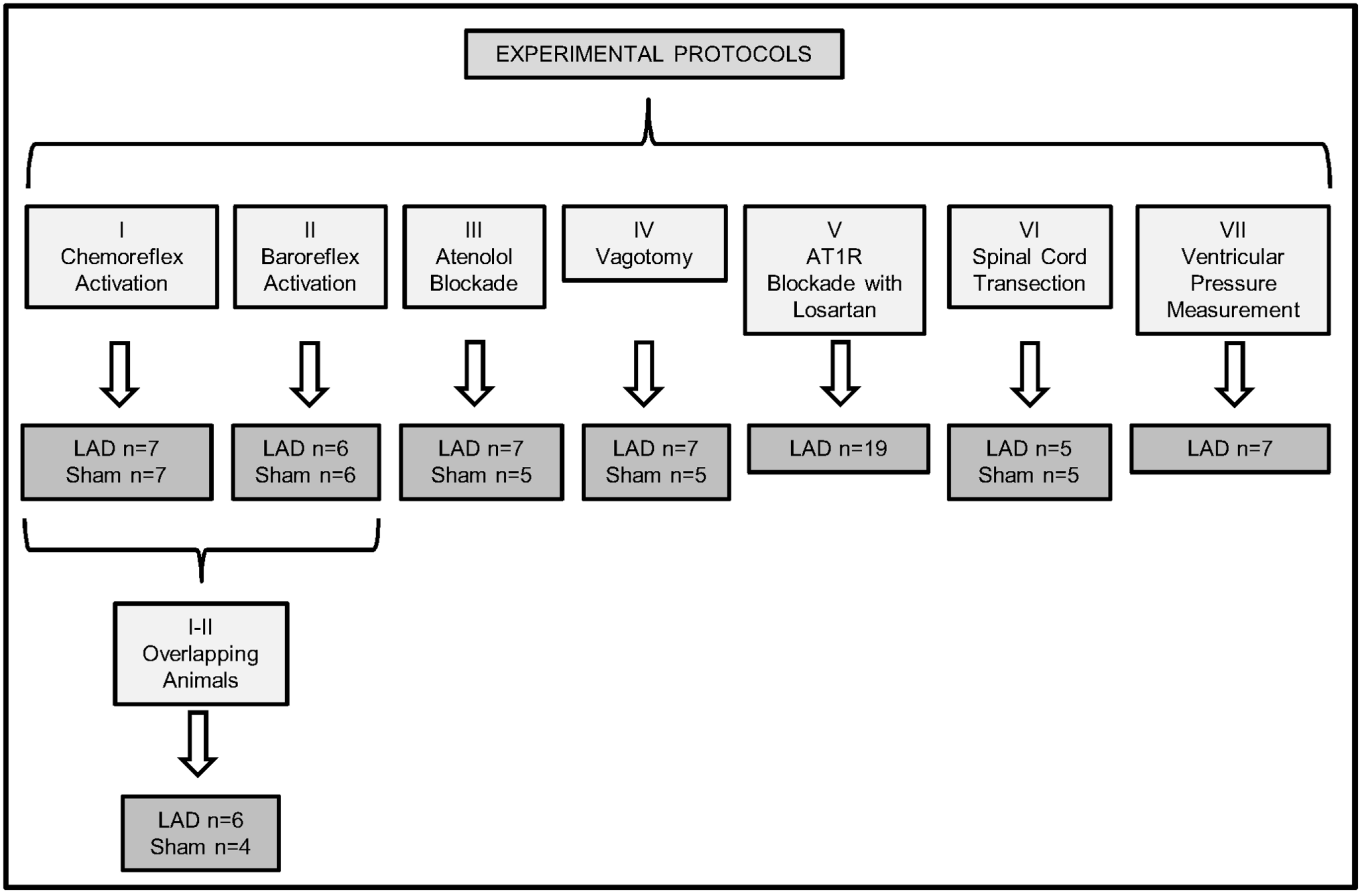
Schematic representation of experimental protocolos performed after LAD ligation or sham surgery. The number of tested animals per group is shown. LAD, left anterior descending coronary artery.

Our aim was to examine changes in respiratory and cardiovascular autonomic tone and its reflex control immediately after coronary ligation (total: n = 76; see Figure 1). Six experimental protocols were performed 60 minutes post LAD ligation and in Sham animals: (I) Peripheral chemoreceptors were stimulated using three doses of sodium cyanide (0.03% solution; 25, 50 and 75 µL bolus) injected into the aorta; being each injection separated by 10 min. The chemoreflex was quantified by measuring the increase in central respiratory rate, the maximum bradycardia and the percentage increase in tSNA during a 5s period at the peak of the chemoreflex sympathetic response compared to an equivalent control period. (II) The baroreceptor reflex was stimulated using phenylephrine (30 µg bolus, i.a., Sigma). The sympathetic (non-cardiac) baroreflex gain was quantified as percentage inhibition of sympathetic activity/Δpressure (%.mmHg^−1^). The percentage of sympathoinhibition was obtained from the ratio of mean tSNA during the peak of the PP increase against the mean activity from a preceding equivalent time period, at an equivalent stage of the respiratory cycle. The bradycardic baroreflex gain was quantified as ΔHR/Δpressure (bpm.mmHg^−1^). (III) Cardiac sympathetic tone was assessed by adding atenolol to the perfusate (20 µg/mL, Sigma) to block cardiac β1-adrenoceptors and inferred from the HR change. (IV) Bilateral cervical vagotomy was performed to measure the cardiac vagal tone as expressed by the HR change. (V) Angiotensin II type 1 receptor (AT1R) antagonist, Losartan potassium (Sigma), was used to evaluate the role of an AT1R in the initiation and maintenance of sympathetic activity post LAD ligation. Either Losartan (40 µM, n = 10) or saline (control, n = 9) was added to the perfusate 1 h and re-applied 2 h post LAD ligation and tSNA recorded up to 3 h after LAD ligation. Half the dose of Losartan used in this study is able to block angiotensin II effects in the mediation of the baroreceptor reflex in the nucleus of the solitary tract [21]. (VI) The spinal cord was fully transected at the first cervical level and the tSNA response measured before (15–20 min) and after (60 min) LAD ligation (n = 5) and in Sham animals (n = 5) to assess contribution of spinal reflexes in generation of sympathetic activity. In a seventh group (Protocol VII), the left ventricular pressure (LVP) was continuously recorded using a PE-10 cannula inserted into the left ventricle via its apex before and post LAD ligation. In the first four protocols (LAD n = 21 and Sham n = 19), basal cardiorespiratory parameters and tSNA were evaluated. The tSNA signal was rectified and integrated (50 ms time constant) and noise levels subtracted before analysis. The mean tSNA (µV) was taken from one minute of recording before (Basal) and at 15, 30, 45 and 60 min post LAD ligation or Sham operated animals. Respiratory-sympathetic activity modulation was evaluated at baseline, 30 and 60 min post LAD ligation and in Sham groups. Phrenic-triggered mean integrated tSNA was carried out and analyzed during: late expiration (Late E), inspiration (I), post-inspiration (PI) and mid-expiration (Mid E). The time duration of Late E, I, PI and Mid E was based on the duration of the inspiratory phrenic burst [22].

### Statistical analysis

All data are expressed as means ± S.E.M. Data were analyzed using one-way ANOVA for repeated measures, followed by Tukey’s post hoc test and Student’s paired and unpaired t-Test with the level of significance set at P<0.05.

## Results

### Area at risk, ECG and hemodynamic changes following coronary ligation in situ

**Figure 2:**
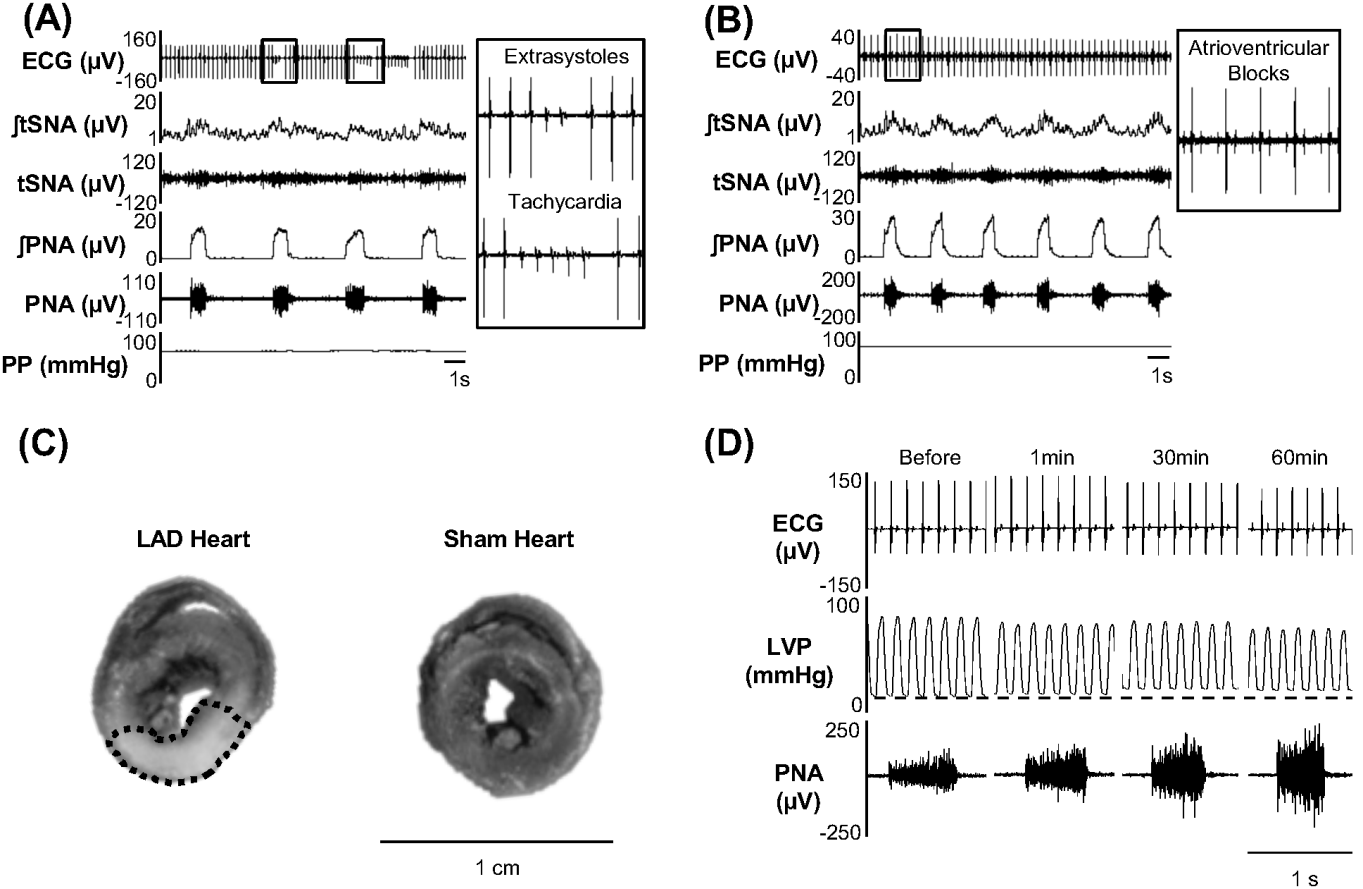
Representative traces from rats showing LVP and transient ECG changes post LAD ligation. (A) Extra-systoles and tachycardia occurred post LAD ligation in some rats. (B) Atrioventricular block starting shortly post LAD ligation. These changes in the ECG lasted 34.0±4.6 min. (C) Median cross-sections of Evans blue dye-stained hearts. The LAD heart cross-section (left) had a safe blue-marked area versus a myocardial area at risk that did not absorb the dye (outlined pale area), contrasting with the Sham heart completely stained blue. (D) Recording showing LVP before and 1, 30, and 60 min post LAD ligation.

The LAD ligation induced transient ECG changes (Figures 2A and 2B) in many preparations (13 of 21 animals) consistent with MI; 11 animals exhibited arrhythmias post ligation and 2 animals showed atrioventricular block. These disturbances lasted up to 34.0±4.6 min when sinus rhythm returned. The myocardial area at risk was 36.3±1.1% of the left ventricle (n = 21, Figure 2C). The LAD ligation led to hemodynamic changes of the left ventricle (n = 7, see Figure 2D and Table 1).

**Table 1:** Hemodynamic parameters before and minutes after LAD ligation in(n = 7).

	Before	1 min	30 min	60 min
LVSP (mmHg)	71.1±5.9	70.6±3.4	69.0±3.5	66.9±3.7
LVEDP (mmHg)	7.5±1.4	13.2±2.0**	14.7±2.6**	14.9±2.4**
LV +dP/dT (mmHg.s^−1^)	1794±173	1559±107*	1530±111*	1460±107**
LV −dP/dT (mmHg.s^−1^)	−1462±187	−1275±133	−1298±141	−1254±140

*P<0.05 and **P<0.01 compared to Before. LVSP, left ventricular systolic pressure; LVEDP, left ventricular end-diastolic pressure; LV dP/dT, maximum rate of LVP rise and fall.

### Baseline changes in cardiorespiratory parameters and tSNA post coronary ligation

**Figure 3:**
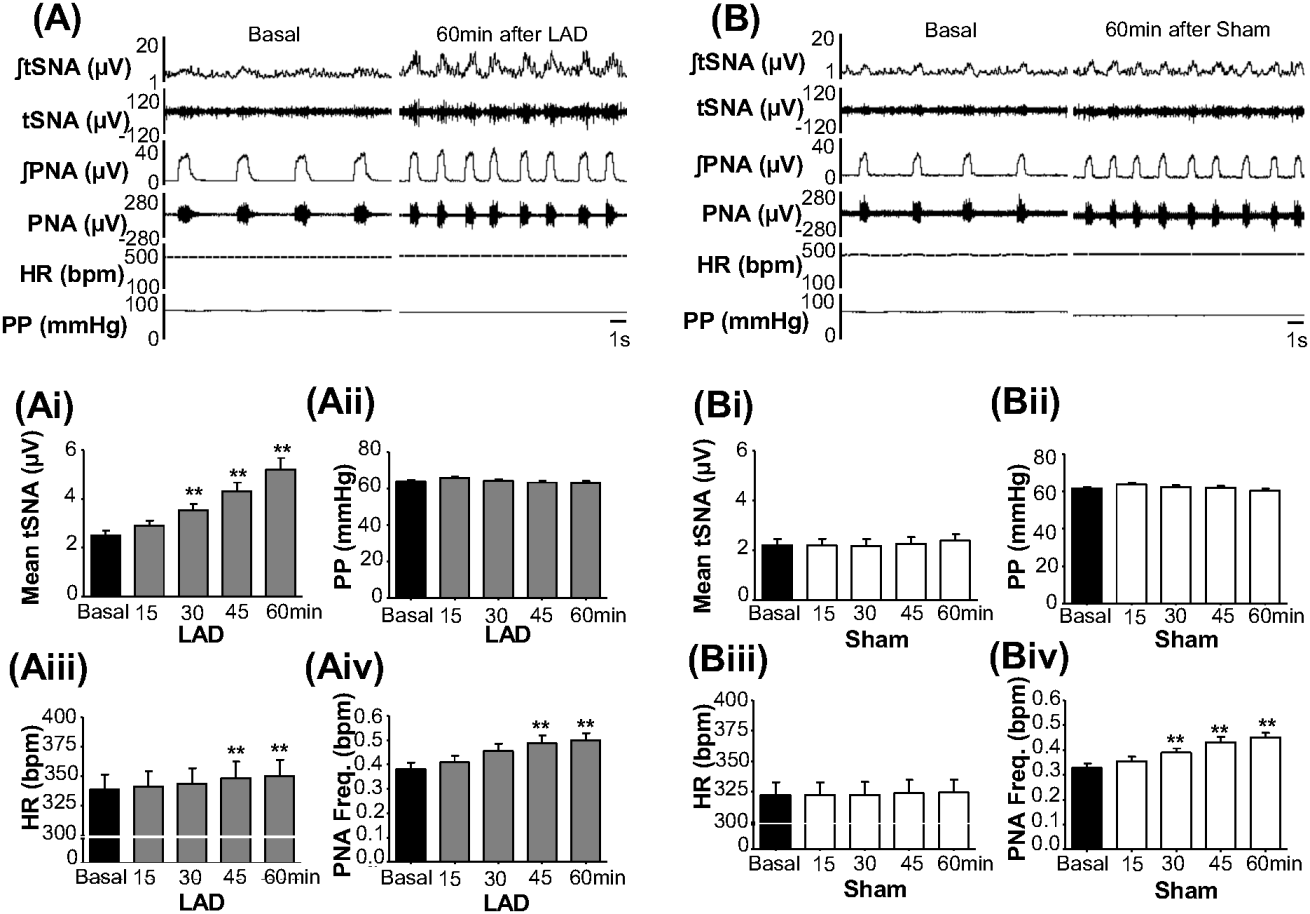
Baseline changes post LAD ligation. Typical traces of raw and integrated tSNA and PNA, ECG and PP before (Basal) and up to 60 min post LAD ligation (A) and in Sham operated (B). Means of tSNA, PP, HR and PNA frequency before (Basal) and at 15, 30, 45 and 60 min post LAD ligation (Ai, Aii, Aiii and Aiv, respectively, n = 21) and Sham operated (Bi, Bii, Biii and Biv, respectively, n = 19). **P<0.01 compared to Basal.

Within 30 min post LAD ligation, mean tSNA increased (45.4±6.7%, reaching 117.2±17.5% by 60 min, P<0.01) while HR increased by 45 min (2.8±0.7%, and 3.3±0.7% at 60 min, P<0.01) [Figures 3 (Ai) and 3 (Aiii); n = 21, P<0.01] compared to Sham rats [Figures 3 (Bi) and 3 (Biii); n = 19, P>0.05]. No changes in PP were found over 60 min post LAD ligation and in Sham rats [Figures 3 (Aii) and 3 (Bii)]; PNA frequency increased in both rat groups to a similar level [Figures 3 (Aiv) and 3 (Biv), P<0.01].

### Peripheral chemoreceptor reflex

**Figure 4:**
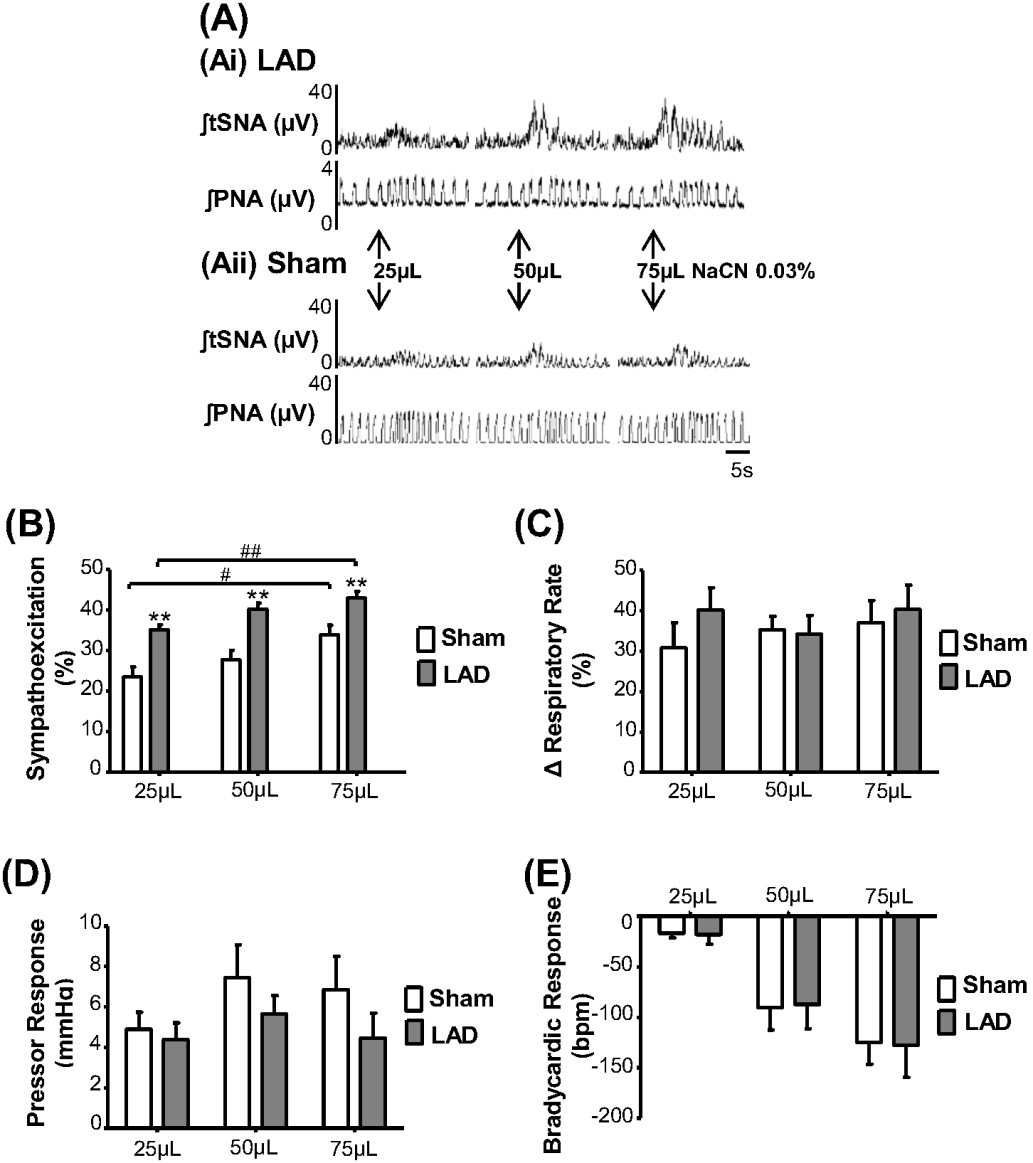
Peripheral chemoreflex responses post LAD ligation. Three doses of sodium cyanide (0.03% solution; 25, 50 and 75 µL, i.a.) were given 60 min post LAD ligation and in Sham operated (n = 7 each group). (A) Recordings of a LAD rat (Ai) and a Sham rat (Aii) showing sympathoexcitation at all doses. (B) Change in sympathoexcitatory response (**P<0.01 compared to Sham). Difference in the magnitude of sympathoexcitation between doses used in the study (^#^P<0.05 and ^##^P<0.01). No changes in chemoreflex evoked responses in respiratory rate (C), pressor (D), and bradycardia (E).

The sympathoexcitatory component of the response to peripheral chemoreceptor activation was greater 60 min post LAD ligation compared to Sham (Figure 4; n = 7 per group, P<0.01, unpaired t-Test) at all doses of cyanide tested (25, 50 and 75 µL: 49.7, 45.3 and 26.8% of increase, respectively). Despite exacerbation of the sympathetic response, there were no differences in peripheral chemoreflex evoked changes in PP, RR or HR.

### Baroreceptor reflex

**Figure 5:**
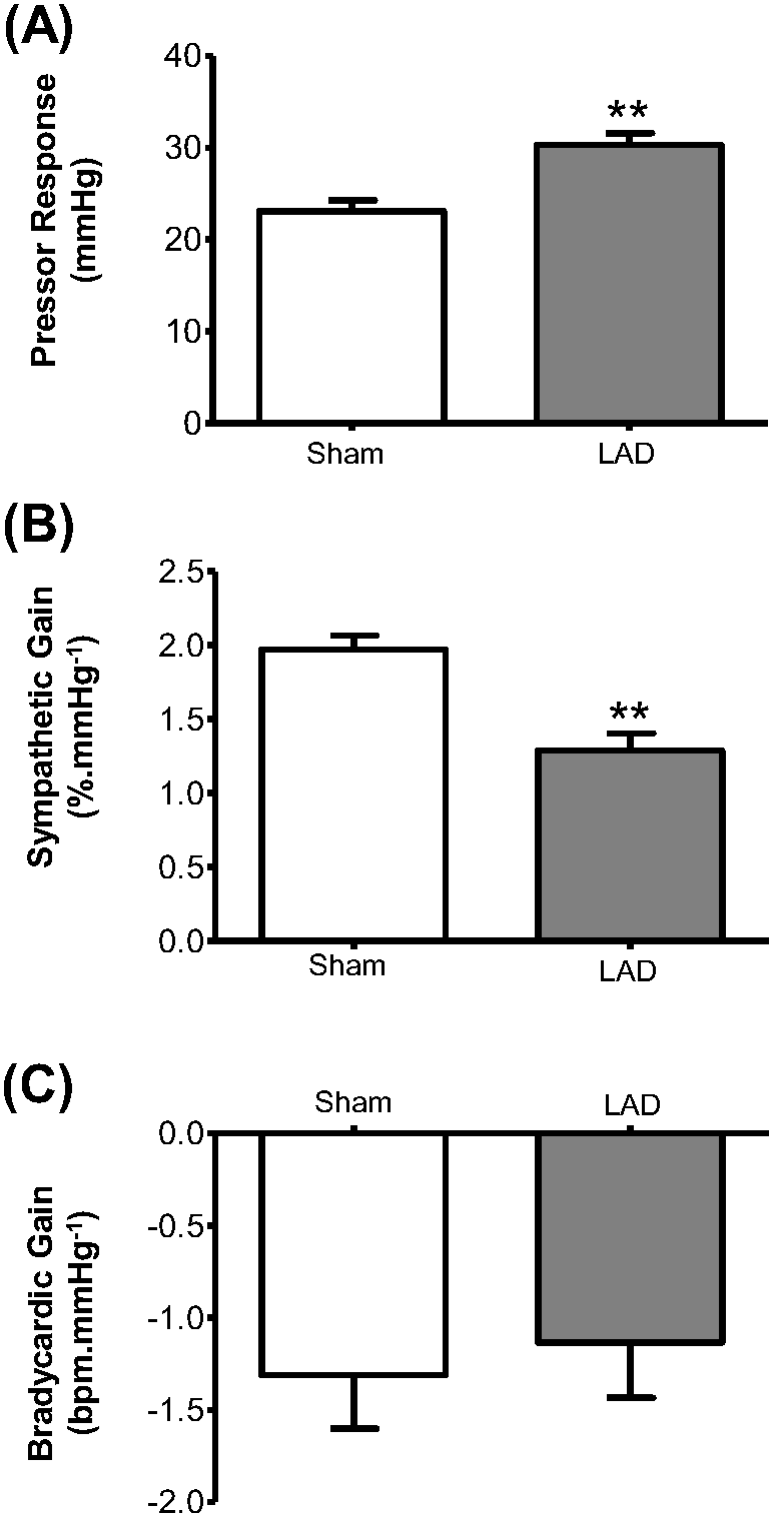
Baroreceptor reflex responses post LAD ligation. Phenylephrine (30 µg, i.a.) was given 60 min post LAD ligation and in Sham rats (n = 6 each group). (A) and (B) Changes in phenylephrine evoked pressor response and sympathetic gain (**P<0.01 compared to Sham). (C) No change in bradycardic gain.

Both the pressor effect and the sympathetic (non-cardiac) baroreflex gain evoked by phenylephrine were significantly different 60 min after LAD ligation versus Sham rats (Figures 5A and 5B; n = 6 each group, P<0.01, unpaired t-Test). The pressor response to phenylephrine was increased (LAD 31.5±1.3 versus Sham 23.1±1.2 mmHg, P<0.01) while the baroreflex gain was depressed (LAD 1.3±0.1 versus Sham 2.0±0.1%.mmHg^−1^; n = 6 per group, P<0.01). The baroreflex bradycardia was unaffected after LAD ligation (Figure 5C).

### Cardiac sympathetic and vagal tones

The cardiac sympathetic tone was elevated post LAD ligation. There was a greater fall in HR in LAD rats compared to Sham group (−107.8±8.0, n = 7, versus −81.6±7.4 bpm, n = 5; P<0.05, unpaired t-Test) after atenolol. Cardiac vagal tone (assessed by bilateral vagotomy) was unaffected by LAD ligation (11.6±1.8, n = 7, versus Sham 13.9±2.4 bpm, n = 5; P>0.05, unpaired t-Test). The level of mean tSNA was also not attenuated by bilateral vagotomy (before LAD ligation: 2.9±0.2; 60 min after LAD ligation/before vagotomy: 4.3±0.3; and 15 min after vagotomy: 7.2±0.6 µV), ruling out a vagal mechanism for sympathoexcitation post LAD ligation.

#### Mechanisms for sympathoexcitation post coronary ligation Angiotensin II

**Figure 6:**
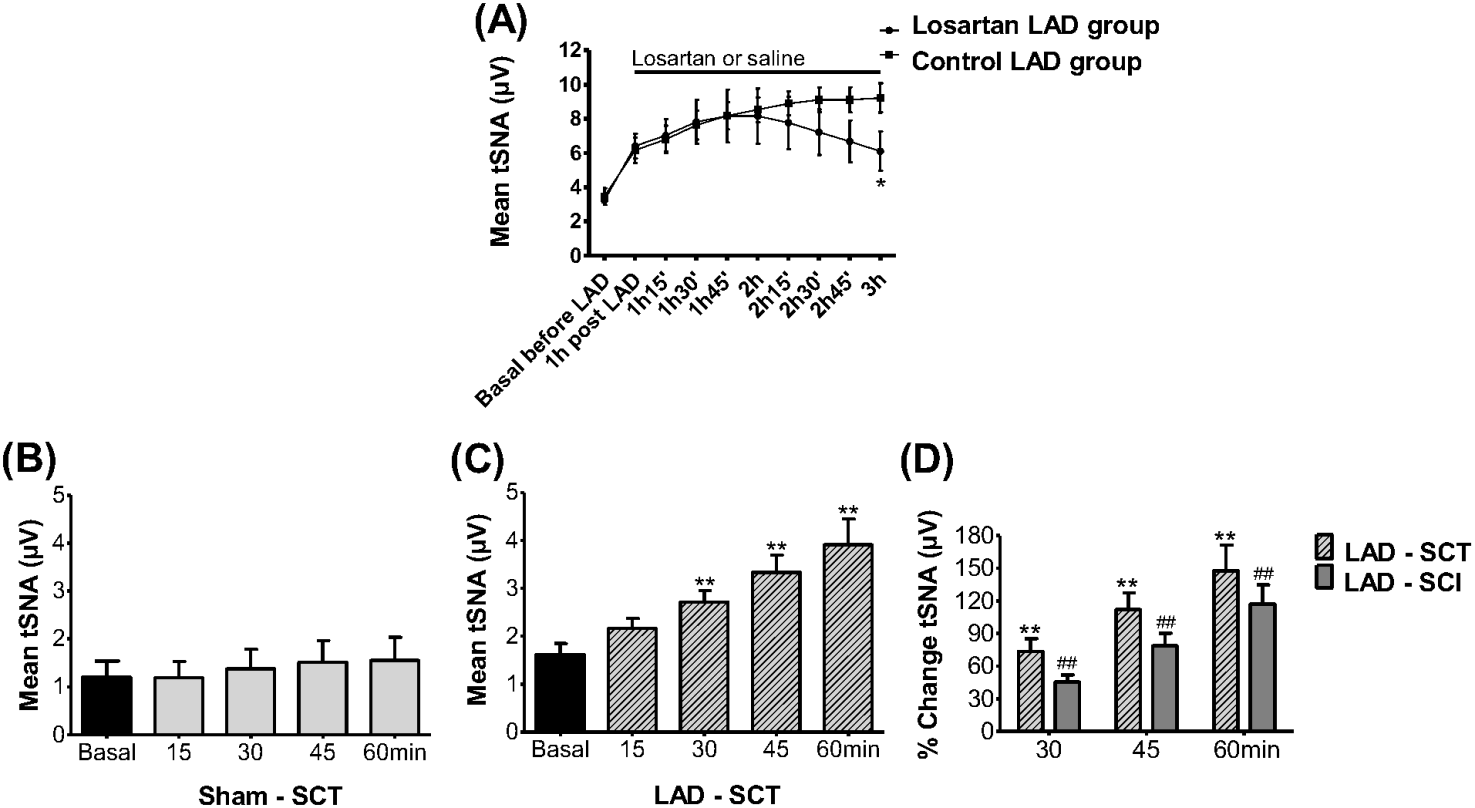
Mechanisms of sympathoexcitation post LAD ligation. (A) The Losartan effect (40 µM added to the perfusate) on the level of tSNA from 1 h to 3 h post LAD ligation (Losartan LAD group: n = 10; Control LAD group: n = 9). The level of mean tSNA was lower at 3 h post LAD ligation in Losartan LAD group (*P<0.05 compared to Control LAD group). (B), (C) and (D) Evaluation of sympathetic activity after spinal cord transection (SCT): Means of tSNA before (Basal) and at 15, 30, 45, and 60 min after LAD ligation (C; n = 5) and in Sham rats (B; n = 5). (D) Percentage of change in mean tSNA at 30, 45 and 60 min after LAD ligation in spinal transected rats (LAD – SCT; n = 5, **P<0.01 compared to respective basal value) and spinal cord intact rats (LAD – SCI; n = 21, ^##^P<0.01 compared to respective baseline). There was no change between the percent increases of both groups (P>0.05).

We assessed whether AT1R were important for the sympathoexcitation post LAD ligation. The mean level of tSNA became different from 3 h onwards (33.8% of difference) between Losartan (n = 10) and Control (n = 9) LAD groups (Figure 6A; *P<0.05, unpaired t-Test) suggesting that an AT1R mediated mechanism was functioning from this time point. The mean level of PP was maintained stable for both groups ruling out any confounding baroreceptor influence.

#### Spinal cord transection

Given that the angiotensinergic mechanism occurred ∼3 h post LAD ligation, we sought other mechanisms that could contribute to the initiation of sympathoexcitation. We assessed whether a spinal mechanism underpinned sympathoexcitation post LAD ligation. In spinal transected rats, tSNA was higher within 30 min post LAD ligation (2.7±0.2 versus basal 1.6±0.2 µV, P<0.01, n = 5; Figure 6C) compared to Sham operated (1.4±0.4 versus basal 1.2±0.3 µV, P>0.05, n = 5; Figure 6B). As seen in the spinal cord intact rats, the increase in mean tSNA continued to climb post LAD ligation with time (i.e. 3.9±0.5 µV by 60 min; P<0.01). In terms of HR, there was a significant drop after spinal transection. Although there was no change in HR at 60 min post LAD ligation in the spinal transected group, it decreased at 60 min in the Sham transected group (P<0.01, see Figure S1 in Supplemental data), suggesting sympathoactivation in the LAD group. The increase in sympathetic activity evoked by LAD ligation at 30, 45, and 60 min of spinal transected rats (LAD: 73.7±11.7, 112.0±15.5, and 147.6±23.9%, respectively; n = 5) was the same as that seen in spinal intact rats (45.4±6.7, 78.7±11.7, and 117.3±17.5%, respectively; n = 21, P>0.05, unpaired t-Test) (Figure 6D).

### Respiratory-sympathetic activity modulation post coronary ligation

Measuring the mean tSNA from four phases of respiratory cycle – late expiration (Late E), inspiration (I), post-inspiration (PI) and mid-expiration (Mid E) 30 and 60 min after LAD ligation (n = 21) and in Sham (n = 19), we observed that tSNA was higher in all expiratory (Late E, Mid E and PI) and inspiratory (I) phases in the LAD group [P<0.01 compared to basal; see Figure S2(A) in Supplemental data], with no changes in Sham group [P>0.05 compared to basal; Figure S2(B)]. The magnitude of these increases was similar between the respiratory phases in LAD group [P>0.05; Figure S2(A)]. These data indicate that the increased strength of respiratory coupling to sympathetic outflow after LAD ligation is non-selective.

## Discussion

The chronic over active sympathetic nervous system post coronary artery ligation has been intensively researched. In contrast, the acute responses of the autonomic nervous system, their onset and mechanisms following MI remain to be well clarified. In the last decades, studies have reported sudden (in the first minute) cardiac afferent and efferent sympathetic responses to transient ischaemia via short-term coronary occlusion in anaesthetized cats [23]–[27] and dogs [28]. However, these studies failed to show sustained activation of afferent discharge and the multiple time-dependent reflex responses and underlying mechanisms mediating these responses in the following minutes post-occlusion. Thus, the present study addresses these deficits of knowledge and provides the first evidence in the rat showing a sustained sympathetic hyperactivity very early following permanent LAD ligation. The thoracic sympathetic activity was increased significantly at 30 min post-occlusion and continued rising over 60 min in our preparation. Although we did not measure the cardiac sympathetic outflow directly, both the increase in basal HR and enhanced bradycardia post atenolol following LAD ligation supports an elevated cardiac sympathetic discharge.

### Timing of autonomic imbalance post coronary ligation

Most experimental studies in humans and animals models have used indirect methods/indices to provide information about changes in the balance of autonomic activity after MI; this reflects the technical difficulty of obtaining direct measurements of autonomic nervous activity. Some studies have investigated plasma catecholamine levels [10], [29]–[32] and norepinephrine spillover [33] in patients with recent MI, HRV in patients weeks/months after MI [1], [34], [35] or baroreflex sensitivity in patients with recent MI (<28 days) [11]. Although these methodologies estimate autonomic activity indirectly, direct measurements of sympathetic nervous activity in animal models are critical to clarify both the precise time course and degree of sympathetic activation post MI as well as investigate the underlying mechanisms.

Graham et al. [2] have obtained directly muscle sympathetic nerve activity (MSNA) measurements at 2 to 4 days, which were repeated at 3 months and 6 months after uncomplicated acute MI in patients. The mean frequency of MSNA was increased 2 to 4 days (first measurement point of the study) post MI and this change lasted for at least 6 months, demonstrating a protracted sympathetic hyperactivity. However, the exact onset of sympathetic over activity was not shown. In contrast, Jardine et al. [36] demonstrated sequential measurement of sympathetic activity before and after (hourly for 3 h and then daily to 7 days) MI in a conscious ovine model. Cardiac sympathetic nerve activity was increased significantly at 60 min following coronary ligation, peaked at 120 min and sustained for 7 days post MI. These data are similar to the time course we observed in the rat. Despite using different experimental approaches, both the latter and the present study were performed in mammals free of the depressant effects of anaesthetics.

### Baro- and chemo- reflex sympathetic responses

In accordance with the increase observed in basal sympathetic activity, the sympathetic components of two major homeostatic reflexes were also altered within 60 min post LAD ligation. We observed a greater chemoreflex evoked sympathoexcitation and depressed sympathetic (non-cardiac) baroreflex gain (i.e. impaired sympathoinhibition), suggesting up-modulation of pro-sympathetic reflex pathways, which is consistent with studies where renal sympathetic nerve activity was measured in pacing-induced chronic heart failure rabbits [37] and heart failure (6–8 weeks after MI) rats [38]; these studies suggest that peripheral chemoreflex sensitivity may be a contributory mechanism to the tonic elevation in sympathetic activity as found in conditions of hypertension [39], [40].

### Cardiac vagal tone and its reflex activation

In contrast to widespread sympathoactivation following LAD ligation, we found that both baseline cardiac vagal tone and reflex bradycardia evoked from both the peripheral chemo- and baro- receptors were unaffected. Variable information is available regarding both tonic and reflex cardiac parasympathetic modulation immediately post infarction. Some studies in humans and cats suggest cardiac vagal over activity in the first minutes/hours following MI [41], [42]. In these studies, vagal over activity was more frequent during inferior infarction whereas sympathetic overactivity occurred in anterior infarction (comparable to coronary ligation of the present study). This may be explained by a preferential distribution of vagal afferents in the inferoposterior wall of the left ventricle that could reflexly drive up cardiac vagal activity. In addition, Lombardi et al. [1] showed sympathetic excitation and a reduced vagal modulation in HR variability (a vagal tone “marker”) at approximately 3 h after MI in humans. We did not confirm any change in vagal tone at 1 h post coronary ligation in the in situ rat. The absence of impaired tonic cardiac vagal activity may be due to our early time evaluation or a species difference. In contrast to our finding, a marked impairment of cardiac baroreflex sensitivity has been well established in patients with chronic heart failure (CHF) [43], [44] but the exact timing of these measurements post MI is not reported. In sum, taking our evidence and that reported previously it appears that immediately after MI cardiac vagal tone is preserved and may increase dependent upon exactly where the infarct has occurred {posterior versus anterior circulation [45]}. This activity may be important in tempering any adverse effects of excessive cardiac sympathetic activity.

### Mechanisms initiating sympathetic activation post coronary ligation

The role of the brain renin-angiotensin II system (RAS) in modulating sympathetic outflow and baroreflex sensitivity [46] in heart disease [47] is well established. Evidence supports that central RAS acting via AT1R modulates sympathetic activity in CHF: (i) Wang & Ma [48] have shown an elevated concentration of angiotensin II in cerebrospinal fluid in dogs with pacing-induced CHF. (ii) AT1R expression is up-regulated in the nucleus of the solitary tract, subfornical organ and paraventricular nucleus after CHF in rats [49] and the rostral ventro-lateral medulla (RVLM) of rabbits after pacing-induced CHF [50]. (iii) Losartan attenuated the AT1R up-regulation in CHF rabbits [50]. (iv) Central blockade of AT1R reduced sympathetic activity and increased baroreflex sensitivity in CHF rabbits [51], [52]. (v) In Wistar rats, both intracerebroventricular infusion and systemic administration of Losartan reduced sympathetic hyperactivity and left ventricular end-diastolic pressure in heart failure induced by coronary artery ligation [16].

Our data revealed that an angiotensinergic mechanism mediated via AT1R underpins the elevation in sympathetic outflow which is functionally detected from 3 h post LAD ligation. Evidence reveals AT1R located in circumventricular organs (eg, subfornical organ), hypothalamic paraventricular nucleus and the supraoptic nucleus are activated following MI and cause sympathoexcitation [53]. Our study was performed in a pre-collicularly decerebrated preparation devoid of the hypothalamic regions, with no functioning blood-brain barrier. Nevertheless, AT1R expression is up-regulated in CHF in the nucleus of the solitary tract [49] and the RVLM [50], [54], [55]; such brainstem areas were preserved and functional in our preparation. Evidence has shown that AT1R up-regulation involves reactive oxygen species in CHF [56]. Furthermore, the existence of brain tissue angiotensin II is also demonstrated by endogenous angiotensinogen synthesis in astrocytes within the medulla oblongata [57]. Thus, we suggest that angiotensinergic activation of brainstem nuclei may be sufficient to sustain sympathetic hyperactivity 3 h after coronary ligation. Certainly, this time course would be consistent with the time for protein synthesis [58] and we assume this to be the case for the AT1R. An increased angiotensinergic mechanism within the NTS could well account for both the elevated peripheral chemoreflex and depressed baroreflex response as we have described before [59]–[61], whereas those in the RVLM could account for the heightened sympathetic discharge.

The initiating mechanism triggering sympathetic over activity after cardiac ischaemia in rats may include a spinal sympathetic cardiocardiac reflex. This reflex was firstly reported by Malliani et al. [25] after coronary ligation in cats. Brown [23] demonstrated that cardiac sympathetic afferent nerve fibres are activated during myocardial ischaemia. Preganglionic sympathetic neurons were shown to become excited post MI, which was sustained after both vagotomy and spinal transection proving a spinal reflex [25]. In our experiments with spinal transection, increased sympathetic activity post LAD ligation was also observed as in intact animals, and HR was maintained and did not fall as seen in the control group, suggesting increased cardiac sympathetic tone. Thus, given the negative data with Losartan at 1 h post LAD ligation, we can propose a non-angiotensinergic spinal mechanism for initiating the increase in sympathetic outflow post coronary ligation.

Besides the cardiac sympathetic afferents, arterial baroreceptor reflex may be altered simultaneously during myocardial ischaemia, because of their unloading triggering reflex increases in efferent sympathetic nerve activity [62]. However, the perfusion pressure was maintained constant during LAD ligation, excluding the role of these receptors in our preparation.

### Functional relevance of prompt sympathetic activation post-MI

The present findings indicate selective up-regulation of sympathetic activity with no change in cardiac vagal tone or reflex modulation. It is reasonable to assume that this initial modulation leads to a protective effect to the heart supporting cardiac function [5]. Although long term sympathetic nervous system activation is detrimental to cardiac function in heart failure [5], [63], in the first moments post MI it compensates for cardiac contractility dysfunction. In addition, increased [64], [65] or preserved vagal control (as found in the present study) in the early phase of acute myocardial ischaemia would exert an antiarrhythmic effect, reducing overall mortality.

In summary, our work shows both increased basal sympathetic drive and chemoreflex sympathoactivation but no changes in vagal regulation in the first hour following coronary artery ligation. Both spinal cord mechanisms and later elevated angiotensinergic activity contribute to increased sympathetic activity generation, some of which is directed to the heart. A detailed knowledge of any mechanisms that are responsible for the autonomic responses following cardiac ischaemia may assist in the temporal design of future effective pharmacological interventions.

## Supporting Information

Figure S1 **HR changes post LAD ligation in spinal animals.** Means of HR before (Basal before SCT) and after (Basal after SCT) spinal cord transection, and in the following 15, 30, 45 and 60 min post LAD ligation (B; LAD – SCT group, n = 5) and in Sham operated (A; Sham – SCT group, n = 5). **P<0.01, *P<0.05 and ^##^P<0.01.
(TIF)

Figure S2 **Respiratory-sympathetic activity coupling.** Phrenic-triggered mean integrated tSNA from four respiratory phases – late expiration (Late E), inspiration (I), post-inspiration (PI) and mid-expiration (Mid E) – before (Basal) and at 30 and 60 min post LAD ligation (A; n = 21) and in Sham (B; n = 19). **^, ##, &&, ††^P<0.01 compared to respective baseline; the magnitude of increase at 30 and 60 min in relation to baseline is given in percentage.
(TIF)
